# Supplementary material for: Gene variations and sweet taste sensitivity in Zambian adults with and without type 2 diabetes mellitus
Source: PLoS One. 2025 Jul 18;20(7):e0328172. doi: 10.1371/journal.pone.0328172 (PMC12273931; doi:10.1371/journal.pone.0328172)
Supplement: S1 Table — (DOCX) [file pone.0328172.s003.docx]

**Supplementary Table 1.** Health variables by combined genotypes in healthy participants and people with Type 2 Diabetes Mellitus.

| Healthy Participants | High Risk | Low Risk | p-value |
| --- | --- | --- | --- |
| No. | 47 | 16 |  |
| Sex (%F) | 52% | 50% |  |
| BMI (kg/m^2^) | 24.6 ± 4.9 | 23.7 ± 4.8 | 0.619 |
| SBP (mmHg) | 115.8 ± 14.6 | 110.0 ± 11.7 | 0.260 |
| DBP (mmHg) | 70.7 ± 11.1 | 68.0 ± 6.3 | 0.580 |
| Pulse (bpm) | 72.9 ± 12.7 | 70.3 ± 13.1 | 0.575 |
| T2DM |  |  |  |
| No. | 27 | 20 |  |
| Sex (%F) | 57% | 85% |  |
| BMI (kg/m^2^) | 27.4 ± 5.8 | 28.7 ± 5.1 | 0.436 |
| SBP (mmHg) | 136.3 ± 21.3 | 133.2 ± 23.5 | 0.639 |
| DBP (mmHg) | 80.3 ± 12.2 | 76.9 ± 14.3 | 0.880 |
| Pulse (bpm) | 88.1 ± 13.5 | 82.9 ± 10.9 | 0.165 |
| Glucose (mmol) | 9.4 ± 4.7 | 9.4 ± 4.7 | 0.579 |

*BMI; body mass index, DBP; diastolic blood pressure, SBP, systolic blood pressure, T2DM; type 2 diabetes mellitus; No.; participant number, Combined Risk; homozygous minor allele and homozygous minor/heterozygous groups, Combined No Risk; homozygous wildtype and homozygous wildtype/heterozygous groups. T-test or Mann Whitney U tests were used where appropriate.*
