# Supplementary material for: Gene variations and sweet taste sensitivity in Zambian adults with and without type 2 diabetes mellitus
Source: PLoS One. 2025 Jul 18;20(7):e0328172. doi: 10.1371/journal.pone.0328172 (PMC12273931; doi:10.1371/journal.pone.0328172)
Supplement: S2 Table — (DOCX) [file pone.0328172.s004.docx]

**Supplementary Table 2.** Disease type by genotype frequency.

| Gene | Genotype | T2DM | Healthy | p-value |
| --- | --- | --- | --- | --- |
| TAS1R3 rs307355 | *^+^*TT | 16 (34.0) | 9 (21.4) | 0.321 |
|  | CT | 16 (34.0) | 20 (47.6) |  |
|  | ***CC | 15 (31.9) | 13 (31.0) |  |
|  | ***CT/TT | 32 (68.1) | 28.8 (69.0) | 0.922 |
|  | *^+^*CC/CT | 31 (66.0) | 33 (78.6) | 0.240 |
| TRPV1 rs4790522 | ***AA | 14 (29.8) | 9 (22.0) | 0.703 |
|  | AC | 21 (48.8) | 20 (48.8) |  |
|  | *^+^*CC | 12 (29.3) | 12 (29.3) |  |
|  | ***AC/CC | 33 (70.2) | 32 (78.0) | 0.471 |
|  | *^+^*AA/AC | 35 (74.5) | 29 (70.7) | 0.811 |
| Combined | Risk | 31 (75.6) | 27 (57.4) | 0.114 |
|  | No Risk | 10 (24.4) | 20 (42.6) |  |

*T2DM; Type 2 Diabetes Mellitus, TAS1R3; Taste 1 Receptor Member 3 gene, rs307355; TT (wildtype), CT, CC, TT/CT (recessive model), CT/CC (dominant model); TRPV1; Transient Receptor Potential Cation Channel Subfamily V Member 1 gene, rs4790522; CC (wildtype), AC, AA, AC/CC (recessive model), AC/CC (dominant model), Combined Risk; homozygous minor allele and homozygous minor/heterozygous groups, Combined No Risk; homozygous wildtype and homozygous wildtype/heterozygous groups *^+^ indicate comparisons made within genotypes. P-value significance at 0.05, significant values are in bold. Chi Squared or Fishers Exact used where appropriate.*
