# Supplementary material for: Gene variations and sweet taste sensitivity in Zambian adults with and without type 2 diabetes mellitus
Source: PLoS One. 2025 Jul 18;20(7):e0328172. doi: 10.1371/journal.pone.0328172 (PMC12273931; doi:10.1371/journal.pone.0328172)
Supplement: Attachment — Submitted filename: PONE-D-24-49147.pdf [file pone.0328172.s005.pdf]

PLOS ONE

TAS1R3 gene variations and sweet taste sensitivity in Africans with and without type 2 diabetes mellitus in Zambia.

--Manuscript Draft--

**Manuscript Number:**	PONE-D-24-49147
**Article Type:**	Research Article
**Full Title:**	TAS1R3 gene variations and sweet taste sensitivity in Africans with and without type 2 diabetes mellitus in Zambia.
**Short Title:**	TAS1R3 gene variations and sweet taste sensitivity
**Corresponding Author:**	Tuku Mwakyoma, MSc<br>Mulungushi University<br>Livingstone, Southern ZAMBIA
**Keywords:**	TAS1R3 genes; sweet taste sensitivity; T2DM
**Abstract:**	Background: Sweet taste perception and preference play crucial roles in dietary habits and health outcomes. Understanding the genetic basis of sweet taste thresholds and preferences can provide insights into individual differences in dietary behavior and susceptibility to metabolic disorders such as type 2 diabetes mellitus (T2DM). In Zambia, there is paucity of data concerning taste perception and preference in relation to genetics among diabetic and non-diabetic individuals. This study aimed to determine the relationship between the TAS1R3 rs307355 genotype, sweet taste thresholds, and diabetic status among adults in Zambia.<br>Methods: A cross-sectional study was conducted among 89 adults at Livingstone University Teaching Hospital (42 non-diabetic and 47 diabetics). Saliva samples were used to determine the TAS1R3 rs307355 genotype. We assessed sweet taste threshold and preference using a series of aqueous sucrose solutions. Demographic characteristics, anthropometrics, lifestyle factors, and dietary habits were collected using a structured questionnaire. Mann-Whitney U tests, Fisher's exact tests, and chi-square tests were the statistical methods used.<br>Results: Diabetic participants were significantly older (median age 56 vs. 29 years, p= 0.001) and had higher BMI (mean 28 vs. 24 p= 0.003) compared to non-diabetic. Diabetic individuals were more likely to be hypertensive (38.3% vs. 7.1%, p= 0.001). Sweet taste thresholds were higher among T2DM participants (17.66 vs. 12.74 g/L, p= 0.040), indicating a low sensitivity to sweet taste. Lastly, there was no relationship between TAS1R3 rs307355 genotype and sweet taste sensitivity in T2DM and non-diabetics.<br>Conclusion: While a correlation was observed between reduced sweet taste sensitivity and T2DM, no genetic link was found between sweet taste sensitivity and TAS1R3 genotype. This suggests that factors beyond genetic variations in TAS1R3 may influence sweet taste perception in individuals with T2DM. However, studies with a larger sample size are required to validate our findings.
**Order of Authors:**	Tuku Mwakyoma, MSc
	Benson M Hamooya, PhD
	Lweendo Muchaili
	Memory Ngosa
	Leta Pilic, PhD
	Sepiso M Masenga, PhD
**Additional Information:**	
**Question**	**Response**
**Financial Disclosure**<br><br>Enter a financial disclosure statement that	Yes

Powered by Editorial Manager® and ProduXion Manager® from Aries Systems Corporation

describes the sources of funding for the work included in this submission. Review the submission guidelines for detailed requirements. View published research articles from *PLOS ONE* for specific examples.<br><br>This statement is required for submission and **will appear in the published article** if the submission is accepted. Please make sure it is accurate.<br><br>**Funded studies**<br>Enter a statement with the following details:<br>• Initials of the authors who received each award<br>• Grant numbers awarded to each author<br>• The full name of each funder<br>• URL of each funder website<br>• Did the sponsors or funders play any role in the study design, data collection and analysis, decision to publish, or preparation of the manuscript?<br><br>Did you receive funding for this work?	
Please add funding details.<br>as follow-up to "**Financial Disclosure**<br><br>Enter a financial disclosure statement that describes the sources of funding for the work included in this submission. Review the submission guidelines for detailed requirements. View published research articles from *PLOS ONE* for specific examples.<br><br>This statement is required for submission and **will appear in the published article** if the submission is accepted. Please make sure it is accurate.	This work was supported by the Hand research group, Mulungushi University, St Mary's University, Twinkenham, London, the Fogarty International Center and National Institute of Diabetes and Digestive and Kidney Diseases of the National Institutes of Health grants R21TW012635 (SKM) and the American Heart Association Award Number 24IVPHA1297559 https://doi.org/10.58275/AHA.24IVPHA1297559.pc.gr.193866 (SKM). The content is solely the responsibility of the authors and does not represent the official views of the National Institutes of Health and the American Heart Association. The funders had no role in the study design, data collection and analysis, decision to publish, or preparation of the manuscript.

**Funded studies**<br>Enter a statement with the following details:<br>• Initials of the authors who received each award<br>• Grant numbers awarded to each author<br>• The full name of each funder<br>• URL of each funder website<br>• Did the sponsors or funders play any role in the study design, data collection and analysis, decision to publish, or preparation of the manuscript?<br><br>Did you receive funding for this work?"	
Please select the country of your main research funder (please select carefully as in some cases this is used in fee calculation).<br>as follow-up to "**Financial Disclosure**<br><br>Enter a financial disclosure statement that describes the sources of funding for the work included in this submission. Review the submission guidelines for detailed requirements. View published research articles from *PLOS ONE* for specific examples.<br><br>This statement is required for submission and **will appear in the published article** if the submission is accepted. Please make sure it is accurate.<br><br>**Funded studies**<br>Enter a statement with the following details:<br>• Initials of the authors who received each award<br>• Grant numbers awarded to each author<br>• The full name of each funder<br>• URL of each funder website<br>• Did the sponsors or funders play any role in the study design, data collection and analysis, decision to publish, or preparation of the manuscript?<br><br>Did you receive funding for this work?"	ZAMBIA - ZM
**Competing Interests**	The authors have declared that no competing interests exist.

Use the instructions below to enter a competing interest statement for this submission. On behalf of all authors, disclose any competing interests that could be perceived to bias this work—acknowledging all financial support and any other relevant financial or non-financial competing interests.<br><br>This statement is required for submission and **will appear in the published article** if the submission is accepted. Please make sure it is accurate and that any funding sources listed in your Funding Information later in the submission form are also declared in your Financial Disclosure statement.<br><br>View published research articles from *PLOS ONE* for specific examples.<br><br>**NO authors have competing interests**<br><br>Enter: *The authors have declared that no competing interests exist.*<br><br>**Authors with competing interests**<br><br>Enter competing interest details beginning with this statement:<br><br>*I have read the journal's policy and the authors of this manuscript have the following competing interests: [insert competing interests here]*<br><br>* typeset	
**Ethics Statement**<br><br>Enter an ethics statement for this submission. This statement is required if the study involved:<br><br>• Human participants<br>• Human specimens or tissue<br>• Vertebrate animals or cephalopods<br>• Vertebrate embryos or tissues	Ethical clearance was sort from Mulungushi University, School of Medicine and Health Sciences Research Ethics Committee (MUHSREC) (Assurance No. FWA0002888 IRB00012281 of IORG0010344) on the 3rd of May, 2023 and the National Health Research Authority (NHRA) on the 23rd August 2023. Permission from Livingstone central Hospital was granted. We provided consent forms to our participants and explained to them, that their participation was voluntary and that they were free to withdraw at any time, without giving a reason and without cost. Written Consent was obtained from our participants by signing the consent form. There was no personal identifying data acquired, therefore participants could not be identified.

• Field research<br><br>Write "N/A" if the submission does not require an ethics statement.<br><br>General guidance is provided below. Consult the submission guidelines for detailed instructions. **Make sure that all information entered here is included in the Methods section of the manuscript.**	

Powered by Editorial Manager® and ProduXion Manager® from Aries Systems Corporation

**Format for specific study types**<br><br>**Human Subject Research (involving human participants and/or tissue)**<br>• Give the name of the institutional review board or ethics committee that approved the study<br>• Include the approval number and/or a statement indicating approval of this research<br>• Indicate the form of consent obtained (written/oral) or the reason that consent was not obtained (e.g. the data were analyzed anonymously)<br><br>**Animal Research (involving vertebrate animals, embryos or tissues)**<br>• Provide the name of the Institutional Animal Care and Use Committee (IACUC) or other relevant ethics board that reviewed the study protocol, and indicate whether they approved this research or granted a formal waiver of ethical approval<br>• Include an approval number if one was obtained<br>• If the study involved *non-human primates*, add *additional details* about animal welfare and steps taken to ameliorate suffering<br>• If anesthesia, euthanasia, or any kind of animal sacrifice is part of the study, include briefly which substances and/or methods were applied<br><br>**Field Research**<br><br>Include the following details if this study involves the collection of plant, animal, or other materials from a natural setting:<br>• Field permit number<br>• Name of the institution or relevant body that granted permission	
**Data Availability**<br><br>Authors are required to make all data underlying the findings described fully available, without restriction, and from the time of publication. PLOS allows rare exceptions to address legal and ethical concerns. See the PLOS Data Policy and FAQ for detailed information.	Yes - all data are fully available without restriction

A Data Availability Statement describing where the data can be found is required at submission. Your answers to this question constitute the Data Availability Statement and **will be published in the article**, if accepted.<br><br>**Important:** Stating 'data available on request from the author' is not sufficient. If your data are only available upon request, select 'No' for the first question and explain your exceptional situation in the text box.<br><br>Do the authors confirm that all data underlying the findings described in their manuscript are fully available without restriction?	
**Describe where the data may be found in full sentences. If you are copying our sample text, replace any instances of XXX with the appropriate details.**<br><br>• If the data are **held or will be held in a public repository**, include URLs, accession numbers or DOIs. If this information will only be available after acceptance, indicate this by ticking the box below. For example: *All XXX files are available from the XXX database (accession number(s) XXX, XXX.*).<br>• If the data are all contained **within the manuscript and/or Supporting Information files**, enter the following: *All relevant data are within the manuscript and its Supporting Information files.*<br>• If neither of these applies but you are able to provide **details of access elsewhere**, with or without limitations, please do so. For example:<br><br>*Data cannot be shared publicly because of [XXX]. Data are available from the XXX Institutional Data Access / Ethics Committee (contact via XXX) for researchers who meet the criteria for access to confidential data.*<br><br>*The data underlying the results presented in the study are available from (include the name of the third party*	The data underlying the results presented in the study have been provided as supporting information S2.

and contact information or URL).<br>• This text is appropriate if the data are owned by a third party and authors do not have permission to share the data.<br><br>* typeset	
Additional data availability information:	

Powered by Editorial Manager® and ProduXion Manager® from Aries Systems Corporation

Manuscript Click here to access/download;Manuscript;Manuscript.docx

TAS1R3 gene variations and sweet taste sensitivity in Africans with and without type 2 diabetes mellitus in Zambia.

Tuku Mwakyoma[1], Benson M. Hamooya[2], Lweendo Muchaili[2], Memory Ngosa[2], Leta Pilic[3], Sepiso K. Masenga[2]

[1]Livingstone University Teaching Hospital Laboratory Department, Livingstone, Zambia

[2]Mulungushi University, School of Medicine and Health Sciences, Livingstone, Zambia

[3]Optimyse Nutrition LTD, United Kingdom.

Emails: Tuku Mwakyoma mwakyomatk@gmail.com – Benson M. Hamooya benmalambo@gmail.com – Lweendo Muchaili muchailil2@gmail.com - Memory Ngosa ngosa.memory@gmail.com – Leta Pilic leta.pilic@gmail.com - Sepiso K. Masenga sepisomasenga@gmail.com

Address for Correspondence:

Tuku Mwakyoma

Livingstone University Teaching Hospital

Laboratory Department

P O Box 60091

Livingstone, Zambia

Tel: +260 978 329000

E-mail: mwakyomatk@gmail.com

Abstract

Background: Sweet taste perception and preference play crucial roles in dietary habits and health outcomes. Understanding the genetic basis of sweet taste thresholds and preferences can provide insights into individual differences in dietary behavior and susceptibility to metabolic disorders such as type 2 diabetes mellitus (T2DM). In Zambia, there is paucity of data concerning taste perception and preference in relation to genetics among diabetic and non-diabetic individuals. This study aimed to determine the relationship between the TAS1R3 rs307355 genotype, sweet taste thresholds, and diabetic status among adults in Zambia.

Methods: A cross-sectional study was conducted among 89 adults at Livingstone University Teaching Hospital (42 non-diabetic and 47 diabetics). Saliva samples were used to determine the TAS1R3 rs307355 genotype. We assessed sweet taste threshold and preference using a series of aqueous sucrose solutions. Demographic characteristics, anthropometrics, lifestyle factors, and dietary habits were collected using a structured questionnaire. Mann-Whitney U tests, Fisher's exact tests, and chi-square tests were the statistical methods used.

Results: Diabetic participants were significantly older (median age 56 vs. 29 years, p= 0.001) and had higher BMI (mean 28 vs. 24 p= 0.003) compared to non-diabetic. Diabetic individuals were more likely to be hypertensive (38.3% vs. 7.1%, p= 0.001). Sweet taste thresholds were higher among T2DM participants (17.66 vs. 12.74 g/L, p= 0.040), indicating a low sensitivity to sweet taste. Lastly, there was no relationship between TAS1R3 rs307355 genotype and sweet taste sensitivity in T2DM and non-diabetics.

Conclusion: While a correlation was observed between reduced sweet taste sensitivity and T2DM, no genetic link was found between sweet taste sensitivity and TAS1R3 genotype. This suggests that factors beyond genetic variations in TAS1R3 may influence sweet taste perception in individuals with T2DM. However, studies with a larger sample size are required to validate our findings.

Key words: TAS1R3 genes; sweet taste sensitivity; T2DM

Introduction

Diabetes is responsible for 1.5 million fatalities per year [1]. In Zambia, it was noted that there were 1.36% fatalities related to Diabetes, with an age-adjusted death rate of 35.64% per 100,000 people [2]. Unhealthy diets are identified as significant risk factors for type 2 diabetes mellitus (T2DM) [3]. High sugar intake was one of the top three dietary risk factors contributing to mortality among T2DM individuals [4]. According to the World Health Organization (WHO), on average, an adult is recommended to take not more than 25 grams of sugar each day[5,6].

Food consumption is influenced by personal experience, dietary habits, hormones, neurotransmitters, and genetic variations[7]. These determine the development of or prevent inadequate body weight management that may lead to diabetes[8]. Diabetes is thought to have a complex aetiology, with genetic, nutritional, physical activity, and environmental variables all playing a role[9]. Dietary sugars have long been thought to raise the risk of T2DM, with a strong biological basis[10]. For example, glucose and maltose generate a quick rise in postprandial glycemia and are independent risk factors for T2DM[11].

Sweet taste sensitivity is defined as the least concentration at which the individual may perceive a given sweet attribute[12] and it may be influenced by genetic differences in the sweet taste receptors such as the taste 1 receptor member 3 (TAS1R3) [13]. According to some research, persons who are less sensitive to sweet food may consume more sugary meals to compensate for their inability to taste them as intensely, this is commonly observed in prediabetes[14] [14]. Individual variations in sweet taste perception have received less attention, with conflicting results[15]. Sweet taste sensitivity has been linked to the genetic ability to perceive prototypical flavour

or stimuli[16]. Sweet flavour has also been connected with sweet food intake and the number of sweet taste receptors (G-protein coupled receptor)[15,17,18]. Excess sugar consumption has been linked to obesity, metabolic disorders, diabetes, cardiovascular disease, cancer, depression, and cognitive impairment[9,19].T2DM is a systematic condition that is particularly prone to these taste disorders[20]. Taste alterations progress with duration of diabetes [21]. People with T2DM tend to consume more sugar than those without it[22].

Taste impairment may be caused by a genetic or acquired taste receptor defect, abnormal brain mechanisms, peripheral neuropathy affecting taste nerves, or microangiopathy affecting taste buds[23]. The exact pathophysiology is unknown. Research suggests that high blood glucose levels can negatively affect taste perception, leading to a cycle of poor glycemic management[24]. Some researchers found no association.

The TAS1R3 coding sequence substantially correlates with human sucrose taste sensitivity and accounts for 16% of population diversity in perception [25]. In this study we selected to explore the variations of the TAS1R3 gene because it encodes the T1R3 receptor protein, which is involved in sweet taste perception[26]. Genetic variations in the TAS1R3 gene can lead to differences in how one perceives sweetness and umami savory tastes [27]. Few studies have analyzed the relation of the allelic diversity of this TAS1R3 gene and sweet taste sensitivity among diabetes and non-diabetes in sub-Saharan africa[28]. Very little is known about the genes in an African population controlling desire for sweet taste, despite the fact that some recent genome wild associated study (GWAS) on understanding the determinants of sweet liking in the African and East Asian ancestry groups in the U.S that was reported[29].

Our goal in this study was to determine whether or not there was a definite genetic relationship between TAS1R3 and sweet taste sensitivity, as well as whether or not those who carried that specific gene had T2DM.

Method

Study design and Subjects

We conducted an analytical cross-sectional study at Livingstone university teaching Hospital among 89 adults of whom 42 and 47 were non-diabetic and diabetic participants, respectively, in the month of September, 2023. All subjects were originally from Livingstone, Zambia. They were informed of the study's purpose and protocol (verbally and in writing). The 42 subjects included hospital health workers and students within the premises that did not have diabetes. The 47 diabetic subjects were recruited during their usual procedures at the Hospital.

Eligibility criteria included participants with or without diabetes mellitus aged ≥18years. This study excluded pregnant women due to altered taste sensitivity that occurs during pregnancy. We also excluded participants with taste disorders (ageusia) and chronic illnesses other than T2DM.

Procedure

Body weight (Kgs) and height (m) were measured without shoes during their examination for the purpose of measuring the body mass index (BMI). BMI was calculated as an individual’s weight in kilograms divided by the square of the height in meters. Demographic data (age, sex) were recorded and assessed together with physical activity, smoking, alcohol habits and whether one had T2DM or not. All

participants were required to test for fasting blood sugar (FBS) to ascertain whether they had T2DM or not.

Sweet taste sensitivity determination sweetness

Sweet taste thresholds were determined as follows; Participants were instructed to avoid eating and drinking (except water) before tasting. Six concentrations (solutions of sucrose in water) of each taste ranging from a “hardly distinguishable” to a “distinct” taste were prepared. Spring water was used as a negative control. The solutions were given in increasing concentrations in each taste series. Following concentrations were used: sucrose in spring water (sweet) in the concentrations 5.0, 15.0, 30.0, 60.0, 120.0 and 240.0 g/L. Participants rinsed their mouth with water for one minute, and then swirled the test solutions in the mouth for approximately 10 seconds before expectorating it. The sweet taste threshold, i.e., the concentration at which the respondent can distinguish the sweet taste from water, and the concentration that a participant preferred, i.e., the concentration the respondents picked as their favorite, were recorded.

Collection of saliva samples and single nucleotide polymorphism (SNP) genotyping

Pure genomic DNA was isolated from the saliva samples (2 ml) provided by the participants. DNA extraction was performed using the commercial kit (PSP® SalivaGene 17 DNA Kit 1011) which is in compliance with European (EU) Directive 98/79/EC on in vitro medical devices. Saliva samples were collected by the research team in Livingstone, Zambia and stored at -20 °C on the premises of the Mulungushi University until shipped to St Mary’s where they were stored at -20 °C and subsequently analyzed. Genetic variation in the taste receptor genes (TAS1R3

rs307355) was analyzed using quantitative real-time polymerase chain reaction (qPCR) technique. Genotyping was done using a technique that was previously reported [30]. Pre-designed TaqMan® SNP genotyping assays for the SNP: rs307335 and the StepOnePlus thermocycler (Applied Biosystems, CA, USA) with two technical replicates for each sample were used. Genotyping call rate was >95%.

Data Analysis Plan

Normality was checked using Shapiro-Wilk test, continuous variables were reported as mean ± SEM or median (interquartile range IQR)). We used the non-parametric test Mann-Whitney to compare medians (interquartile range) of continuous variables between non-diabetes and diabetes. For categorical variables, we employed Chi-square or Fisher's exact test, as applicable. Allele carriers were TT, TC and CC for TAS1R3 rs307355 of single nucleotide polymorphisms. The association between sweet taste threshold among individuals with or without T2DM were obtained by conducting a t-test.

Ethical approval

Ethical clearance was sort from Mulungushi University, School of Medicine and Health Sciences Research Ethics Committee (MUHSREC) (Assurance No. FWA0002888 IRB00012281 of IORG0010344) on the 3rd of May, 2023 and the National Health Research Authority (NHRA) on the 23rd August 2023. Permission from Livingstone central Hospital was granted. We provided consent forms to our participants and explained to them, that their participation was voluntary and that they were free to withdraw at any time, without giving a reason and without cost. Written Consent was obtained from our participants by signing the consent form. There was no personal identifying data acquired, therefore participants could not be identified.

Results

Participants characteristics

The participants had a median age of 46 (IQR 28,59). Diabetic participants were older (median age 56 vs. 29 years), had a higher BMI (mean 27.98 vs. 24.53), majority were hypertensive (38.3% vs.7.1%), had higher sucrose threshold (17.66g/L vs.12.74g/L). Participants with T2DM had a higher threshold of taste sensitivity. Diabetics preferred a lower sucrose concentration (39.15g/L vs 74.40g/L) compared to those without diabetes. Table 1. Participants were African (100%) and primarily non-smokers (97.8%).

Table 1. Participant characteristics

**Variables**	**Frequency or median (Percentage or IQR)**	**Diabetics**	**Non-Diabetes**	**P-Value**
**Age,** *years*	46 (28,59)	56 (52, 60)	29 (28, 34)	**<0.001**
**Sex**				
Male	36 (40.4)	15 (31.9%)	21 (50%)	0.083
Female	53 (59.6)	32 (68.1%)	21 (50%)	
**Do you smoke?**				
No	87 (97.8)	47 (100%)	40 (95.2%)	0.13
Yes	2 (2.2)	0 (0%)	2 (4.8%)	
**Do you drink any alcoholic beverages?**				
No	72 (80.9)	42 (89.4%)	30 (71.4%)	**0.032**
Yes	17 (19.1)	5 (10.6%)	12 (28.6%)	
**During a typical week approximately how, many hours do you spend exercising?**	0.5 (0.0, 2.0)	0.50 (0.86, 1.92)	0.75 (0.95, 2.72)	0.693
**BMI,** *kg/m^2, mean(SD)*	26.4 (5.4)	27.98 (5.52)	24.53 (4.90)	**0.003**
**Hypertension**				
No	68 (76.4)	29 (61.7)	39 (92.9)	**0.001**
Yes	21 (23.6)	18 (38.3)	3 (7.1)	
**Genotype**				

TT	25 (28.1)	16 (34.0)	9 (21.4)	0.321
TC	36 (40.4)	16 (34.0)	20 (47.6)	
CC	28 (31.5)	15 (31.9)	13 (31.0)	
**Are you currently following a specific diet for medical reasons?**				
No	67 (75.3)	26 (55.3%)	41 (97.6%)	**<0.001**
Yes	22 (24.7)	21 (44.7%)	1 (2.4%)	
**Sucrose concentration (g/L) preferred**	55.8 (52.4)	74.40	39.15	**0.001**
**Sweet taste threshold (g/L) (mean) SD**	15.3 (11.3)	17.66	12.74	**0.040**

The genetic basis of sweet taste threshold and preference

There was no significant difference in thresholds or sweet taste preference between the TAS1R3 rs307355 genotype groups.

Table 2. Key outcome variables for the TAS1R3 rs307355 genotype

**Variables**	**rs307355**			
	**TT n=25**	**TC n=36**	**CC n=28**	**P-Value**
**Do you smoke?**				
No	25 (100)	36 (100)	26 (92.9)	0.108
Yes	0	0	2 (7.1)	
**Sucrose concentration (g/L) preferred**	36.60	48.17	48.43	0.141
**Sucrose concentration (g/L) threshold (sweet taste threshold)**	42.68	40.83	52.43	0.120

We found no link between genetics and sweet taste perception. The **Figure 1** depicts the relationship between genetics and the sweet taste threshold, specifically

examining how the number of participants varies with the actual sucrose concentration (g/L) threshold based on their genotype.

Figure 1: Genetics and sweet taste sensitivity. The three genotypes represented are: **TT** (blue line), **TC** (green line) and **CC** (yellow-brown line). The x-axis represents the actual sucrose concentration in grams per liter (g/L), which seems to serve as a threshold for sweet taste sensitivity. The y-axis represents the number of participants corresponding to each genotype. TC genotype reaches the highest peak, showing the largest number of participants (about 20) at a sucrose concentration of around 15 g/L. CC genotype follows a similar pattern but peaks slightly lower than the TC genotype. TT genotype remains relatively flat compared to the other two, with fewer participants across the sucrose concentrations. We found that individuals with the TC genotype may be more sensitive to sweetness, as more participants reach their threshold at a lower sucrose concentration. Conversely, individuals with the TT genotype appear to have a higher threshold, requiring higher sucrose concentrations to detect sweetness.

Additionally, we found no link between genetics and sweet taste preference. Figure 2 simply shows the sucrose preference at different concentrations chosen by participants with different alleles. The figure 2 depicts the relationship between genetics and sweet taste preference, exploring how the number of participants in each genotype group correlates with their preferred sucrose concentration (g/L).

Figure 2: Genetics and sweet taste preference. The three genotypes represented are: **TT** (blue line), **TC** (green line) and **CC** (yellow-brown line). The x-axis represents the actual sucrose concentration in grams per liter (g/L) that participants preferred. The y-axis represents the number of participants corresponding to each genotype. TC genotype shows the highest peak, with around 12 participants preferring a sucrose concentration near 30 g/L. The line gradually decreases as sucrose concentration increases. CC genotype, the peak for this group occurs slightly before the TC group, with around 10 participants preferring concentrations close to 30 g/L. The number of participants then drops off more slowly at higher concentrations. TT genotype participants, prefer a wider range of sucrose concentrations, with a peak of 10 participants around 30 g/L, followed by a rapid decrease as the sucrose concentration

increases. This figure suggests that participants with the TC genotype may have a stronger preference for moderate sucrose concentrations, whereas those with the TT genotype exhibit a broader range of sucrose preferences. The CC genotype group appears to have preferences similar to the TC group but is less pronounced at higher concentrations.

Discussion

The purpose of this study was to look at the links between sweet taste receptor (TAS1R3), sweet taste sensitivity (threshold sensitivity), in people with and without T2DM.

There was an association between diabetes and a higher threshold for sucrose concentrations, T2DM were less likely to perceive sucrose and had low sweet sensitivity. Our findings of low sweet taste sensitivity in T2DM are consistent with findings of previous studies conducted[24,31]. This diminished sensitivity to sweetness may contribute to altered dietary preferences and behaviors among individuals with diabetes, potentially influencing their overall glycemic control and metabolic health [23,31–33]. Healthcare providers involved in the management of T2DM should therefore take into account this altered taste perception as it has potential to negate planned dietary modification plans, and make effort to educate these patients on its further implications on diabetes. Increased education and awareness among diabetics on taste perception alteration and need for well-planned dietary modifications can improve their dietary choices, hence improving their overall health outcomes [34]. We also found that the T2DM participants in this study preferred lower concentration sucrose similar to a study that was conducted in Korea[35]. The

majority of our T2DM admitted to being on a diet, however the primary cause of the decreased preference was not investigated.

Our study found no association between TAS1R3 variations and sweet taste perception and identification thresholds. However, since our study had a small sample size (n=89), it is possible that this could have influenced the study outcome as smaller sample sizes are prone to random error. Additionally, differences in taste perception testing methodologies between different studies [36,37] could explain the discrepancy in results. Studying different sweet taste receptor genetic variations could have contributed to a more accurate conclusion and linkage, given the paucity of knowledge regarding taste receptor genes in the African population. Furthermore, age range disparities between research, as well as differences in study participants, may account for the discrepancies in results. The fact that no correlation between the SNP and thresholds was found in the current study does not imply that TAS1R3 has no effect on sweet taste thresholds[18]. A similar study conducted among a European population discovered that individual with alleles TC, SNPs located upstream of the TAS1R3 coding region, rs307355 and rs35744813, are highly related to human taste sensitivity to sucrose and individuals with T alleles are less susceptible to sucrose than people with C alleles [25]. A different study found that TAS1R3-linked polymorphic genotypes with heterozygous, TC, alleles at rs307355 are related to strong sweet taste sensitivity and that diabetes had a higher preference for sweetness [38].

The African population has a scarcity of data on taste sensitivity and taste receptors. This study will play a role as a baseline study to show genetic differences in sweet taste sensitivity in a T2DM population in sub-Saharan Africa. The findings of this study indicate a link between high sweet taste sensitivity and T2DM. The findings in this

research also provide some insight into the genetic distribution of alleles in black Africans.

Our current study has some notable limitations. In contrast to the sample utilized in this investigation, large sample sizes are typically required to adequately detect small genetic connections. The actual sugar intake was not measured among the participants. Real -world eating experiences, on the other hand, are influenced by aspects that cannot be mimicked in lab settings, such as social environment, emotional state, and overall meal composition. One of the main risk factors for prediabetes and diabetes is advanced age. Age range restrictions were necessary to prevent comparisons between very old people and young adults. Lastly, conducting a long-term study on sweet taste receptors may provide insights into the risk of developing T2DM.

Conclusions

Participants with T2DM had lower taste sensitivity. Despite their lower perception of sucrose, T2DM participants show a lesser desire for sweet tastes than non-diabetic participants. The genetic diversity of sweet taste receptor genes may help us better understand individual differences in metabolic disease risk, such as obesity and diabetes. More research is needed to establish how these SNPs affect taste sensitivity and how they relate to metabolism.

Availability of data and materials

The data underlying the results presented in the study have been provided as supporting information S2.

Competing interests

The authors declare no competing interests. Leta Pilic is founder of Optimyse Nutrition, company providing nutrigenetic testing for individuals and health professionals.

Funding

This work was supported by the Hand research group, Mulungushi University, St Mary's University, Twinkenham, London, the Fogarty International Center and National Institute of Diabetes and Digestive and Kidney Diseases of the National Institutes of Health grants R21TW012635 (SKM) and the American Heart Association Award Number 24IVPHA1297559 https://doi.org/10.58275/AHA.24IVPHA1297559.pc.gr.193866 (SKM). The content is solely the responsibility of the authors and does not represent the official views of the National Institutes of Health and the American Heart Association. The funders had no role in the study design, data collection and analysis, decision to publish, or preparation of the manuscript.

Author's contributions

LP, SKM, and TM designed the research. SKM, BMH, TM, LM, and NG all helped write the manuscript. TM, LM, and SKM gathered data. SKM, BMH, and TM carried out data validation, visualization, and analysis. TM is the lead investigator. SKM is the lead author and guarantor. All writers read, provided feedback on, and approved the final manuscript.

Acknowledgments

We are very grateful to all Laboratory personnel at St Mary's University, the Senior Medical Superintendent's Livingstone University Teaching Hospital (LUTH) for their support. And lastly, we would to thank Mulungushi University for the support

References

1. Diabetes - PAHO/WHO | Pan American Health Organization. 14 Nov 2023 [cited 3 Mar 2024]. Available: https://www.paho.org/en/topics/diabetes

2. Nyirongo S, Mukwato PK, Musenge EM, Kalusopa VM. Adherence to Treatment by Patients with Type 2 Diabetes Mellitus at Monze Mission Hospital, Monze, Zambia. Open J Nurs. 2021;11: 184–203. doi:10.4236/ojn.2021.113017

3. Dika H, Deogratias M, Byamungu D, Marwa K, Kapesa A, Mwita S. Practice and Knowledge on Type 2 Diabetes Mellitus Risk Factors Among Office Workers in Mwanza City, Tanzania. East Afr Health Res J. 2023;7: 81–87. doi:10.24248/eahrj.v7i1.712

4. Ramne S, Alves Dias J, González-Padilla E, Olsson K, Lindahl B, Engström G, et al. Association between added sugar intake and mortality is nonlinear and dependent on sugar source in 2 Swedish population–based prospective cohorts. Am J Clin Nutr. 2019;109: 411–423. doi:10.1093/ajcn/nqy268

5. WHO guideline : sugar consumption recommendation. In: WHO guideline : sugar consumption recommendation [Internet]. 3 Apr 2015 [cited 14 Jun 2024]. Available: https://www.who.int/news/item/04-03-2015-who-calls-on-countries-to-reduce-sugars-intake-among-adults-and-children

6. Yan RR, Chan CB, Louie JCY. Current WHO recommendation to reduce free sugar intake from all sources to below 10% of daily energy intake for supporting overall health is not well supported by available evidence. Am J Clin Nutr. 2022;116: 15–39. doi:10.1093/ajcn/nqac084

7. Grimm ER, Steinle NI. Genetics of Eating Behavior: Established and Emerging Concepts. Nutr Rev. 2011;69: 52–60. doi:10.1111/j.1753-4887.2010.00361.x

8. Galindo MM, Schneider NY, Stähler F, Töle J, Meyerhof W. Taste preferences. Prog Mol Biol Transl Sci. 2012;108: 383–426. doi:10.1016/B978-0-12-398397-8.00015-0

9. Liu Y, Cheng J, Wan L, Chen W. Associations between Total and Added Sugar Intake and Diabetes among Chinese Adults: The Role of Body Mass Index. Nutrients. 2023;15: 3274. doi:10.3390/nu15143274

10. Huang Y, Chen Z, Chen B, Li J, Yuan X, Li J, et al. Dietary sugar consumption and health: umbrella review. The BMJ. 2023;381: e071609. doi:10.1136/bmj-2022-071609

11. Bonora E, Muggeo M. Postprandial blood glucose as a risk factor for cardiovascular disease in Type II diabetes: the epidemiological evidence. Diabetologia. 2001;44: 2107–2114. doi:10.1007/s001250100020

12. Bertoli S, Laureati M, Battezzati A, Bergamaschi V, Cereda E, Spadafranca A, et al. Taste sensitivity, nutritional status and metabolic syndrome: Implication in weight loss dietary interventions. World J Diabetes. 2014;5: 717–723. doi:10.4239/wjd.v5.i5.717

13. Bachmanov AA, Bosak NP, Floriano WB, Inoue M, Li X, Lin C, et al. Genetics of sweet taste preferences. Flavour Fragr J. 2011;26: 286–294. doi:10.1002/ffj.2074

14. Keskitalo K, Knaapila A, Kallela M, Palotie A, Wessman M, Sammalisto S, et al. Sweet taste preferences are partly genetically determined: identification of a trait locus on chromosome 16. Am J Clin Nutr. 2007;86: 55–63. doi:10.1093/ajcn/86.1.55

15. Melis M, Mastinu M, Naciri LC, Muroni P, Tomassini Barbarossa I. Associations between Sweet Taste Sensitivity and Polymorphisms (SNPs) in the TAS1R2 and TAS1R3 Genes, Gender, PROP Taster Status, and Density of Fungiform Papillae in a Genetically Homogeneous Sardinian Cohort. Nutrients. 2022;14: 4903. doi:10.3390/nu14224903

16. Reed DR, McDaniel AH. The Human Sweet Tooth. BMC Oral Health. 2006;6: S17. doi:10.1186/1472-6831-6-S1-S17

17. Jayasinghe SN, Kruger R, Walsh DCI, Cao G, Rivers S, Richter M, et al. Is Sweet Taste Perception Associated with Sweet Food Liking and Intake? Nutrients. 2017;9: 750. doi:10.3390/nu9070750

18. Han P, Keast R, Roura E. TAS1R1 and TAS1R3 Polymorphisms Relate to Energy and Protein-Rich Food Choices from a Buffet Meal Respectively. Nutrients. 2018;10: 1906. doi:10.3390/nu10121906

19. The Impact of Free Sugar on Human Health—A Narrative Review - PMC. 2023 [cited 14 Jun 2024]. Available: https://www.ncbi.nlm.nih.gov/pmc/articles/PMC9966020/

20. Al-Maskari AY, Al-Maskari MY, Al-Sudairy S. Oral Manifestations and Complications of Diabetes Mellitus. Sultan Qaboos Univ Med J. 2011;11: 179–186.

21. Bhandare NN, Keny MS, Nevrekar RP, Bhandare PN. Diabetic Tongue - Could it be a Diagnostic Criterion? J Fam Med Prim Care. 2014;3: 290–291. doi:10.4103/2249-4863.141654

22. Basu S, Yoffe P, Hills N, Lustig RH. The Relationship of Sugar to Population-Level Diabetes Prevalence: An Econometric Analysis of Repeated Cross-Sectional Data. PLoS ONE. 2013;8: e57873. doi:10.1371/journal.pone.0057873

23. Catamo E, Robino A, Tinti D, Dovc K, Franceschi R, Giangreco M, et al. Altered Taste Function in Young Individuals With Type 1 Diabetes. Front Nutr. 2022;8: 797920. doi:10.3389/fnut.2021.797920

24. Pugnaloni S, Alia S, Mancini M, Santoro V, Di Paolo A, Rabini RA, et al. A Study on the Relationship between Type 2 Diabetes and Taste Function in Patients with Good Glycemic Control. Nutrients. 2020;12: 1112. doi:10.3390/nu12041112

25. Fushan AA, Simons CT, Slack JP, Manichaikul A, Drayna D. Allelic polymorphism within the TAS1R3 promoter is associated with human taste sensitivity to sucrose. Curr Biol CB. 2009;19: 1288–1293. doi:10.1016/j.cub.2009.06.015

26. Inoue M, Glendinning JI, Theodorides ML, Harkness S, Li X, Bosak N, et al. Allelic variation of the Tas1r3 taste receptor gene selectively affects taste responses to sweeteners: evidence from 129.B6-Tas1r3 congenic mice. Physiol Genomics. 2007;32: 82–94. doi:10.1152/physiolgenomics.00161.2007

27. Farinella R, Erbi I, Bedini A, Donato S, Gentiluomo M, Angelucci C, et al. Polymorphic variants in Sweet and Umami taste receptor genes and birthweight. Sci Rep. 2021;11: 4971. doi:10.1038/s41598-021-84491-4

28. Fushan AA, Simons CT, Slack JP, Manichaikul A, Drayna D. Allelic Polymorphism within the TAS1R3 Promoter is Associated with Human Taste Sensitivity to Sucrose. Curr Biol CB. 2009;19: 1288–1293. doi:10.1016/j.cub.2009.06.015

29. Cheung MM, Hubert PA, Reed DR, Pouget ER, Jiang X, Hwang L-D. Understanding the Determinants of Sweet Liking in the African and East Asian Ancestry Groups in the U.S. – A Study Protocol. Res Sq. 2023; rs.3.rs-3644422. doi:10.21203/rs.3.rs-3644422/v1

30. Pilic L, Mavrommatis Y. Genetic predisposition to salt-sensitive normotension and its effects on salt taste perception and intake. Br J Nutr. 2018;120: 721–731. doi:10.1017/S0007114518002027

31. Wasalathanthri S, Hettiarachchi P, Prathapan S. Sweet taste sensitivity in pre-diabetics, diabetics and normoglycemic controls: a comparative cross sectional study. BMC Endocr Disord. 2014;14: 67. doi:10.1186/1472-6823-14-67

32. Paglia L. The sweet danger of added sugars. Eur J Paediatr Dent. 2019;20: 89. doi:10.23804/ejpd.2019.20.02.01

33. Sińska BI, Kucharska A, Czarnecka K, Harton A, Szypowska A, Traczyk I. Sensitivity to Sweet and Salty Tastes in Children and Adolescents with Type 1 Diabetes. Nutrients. 2022;15: 172. doi:10.3390/nu15010172

34. Minari TP, Tácito LHB, Yugar LBT, Ferreira-Melo SE, Manzano CF, Pires AC, et al. Nutritional Strategies for the Management of Type 2 Diabetes Mellitus: A Narrative Review. Nutrients. 2023;15: 5096. doi:10.3390/nu15245096

35. Yu JH, Shin M-S, Lee JR, Choi JH, Koh EH, Lee WJ, et al. Decreased sucrose preference in patients with type 2 diabetes mellitus. Diabetes Res Clin Pract. 2014;104: 214–219. doi:10.1016/j.diabres.2014.02.007

36. Mojet J. Taste perception with age. Wageningen: Ponsen en Looijen; 2004.

37. Okayama T, Watanabe H. Association between taste perception, nutrient intake, and mental health in young Japanese women. Nutr Res Pract. 2019;13: 41–46. doi:10.4162/nrp.2019.13.1.41

38. Diószegi J, Llanaj E, Ádány R. Genetic Background of Taste Perception, Taste Preferences, and Its Nutritional Implications: A Systematic Review. Front Genet. 2019;10: 1272. doi:10.3389/fgene.2019.01272

Supporting information

S1 Strobe checklist

S2 Dataset

Figure 1

Click here to access/download;Figure;Figure 1.tif

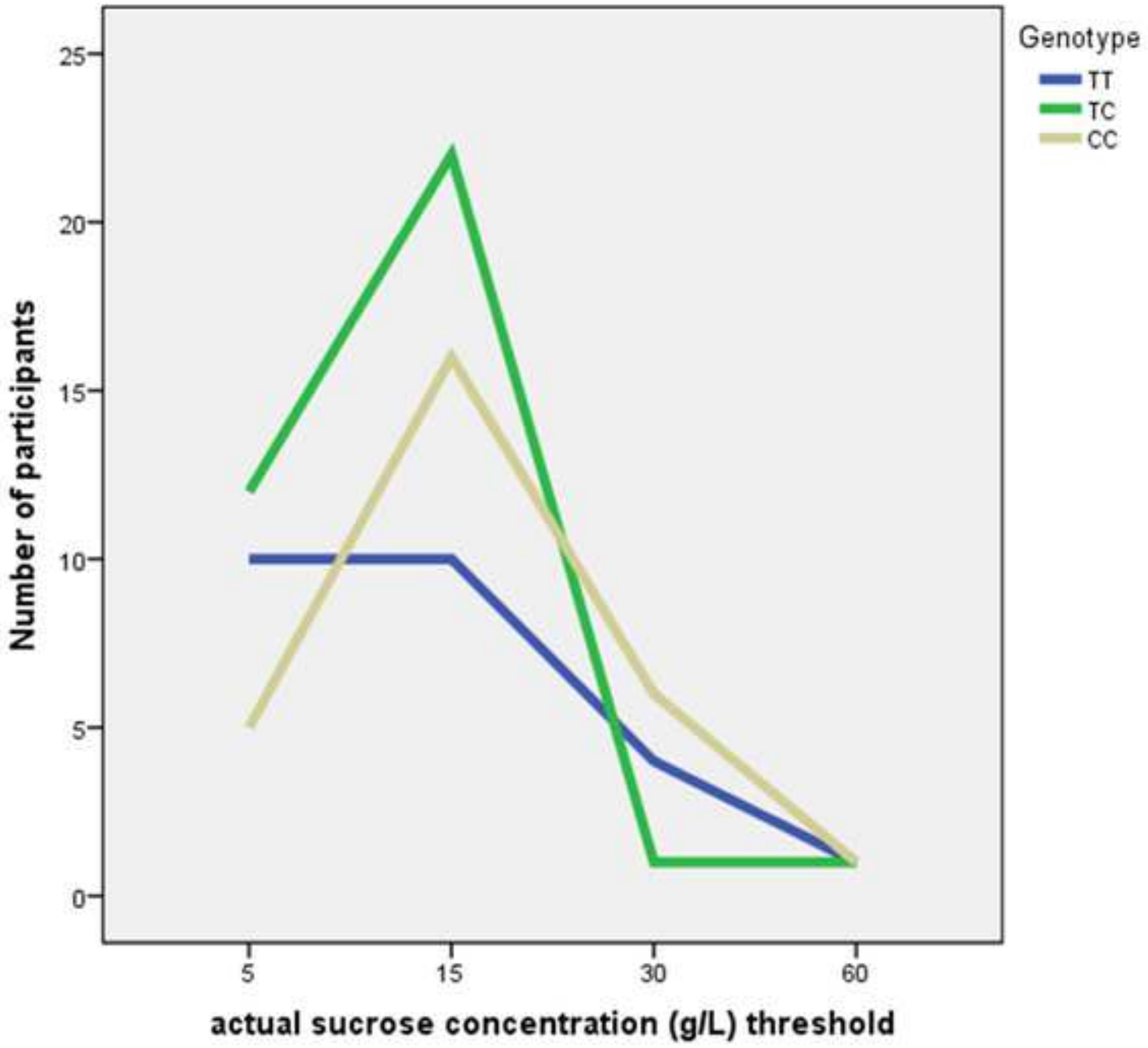

Figure 2

Click here to access/download;Figure;figure 2.tif

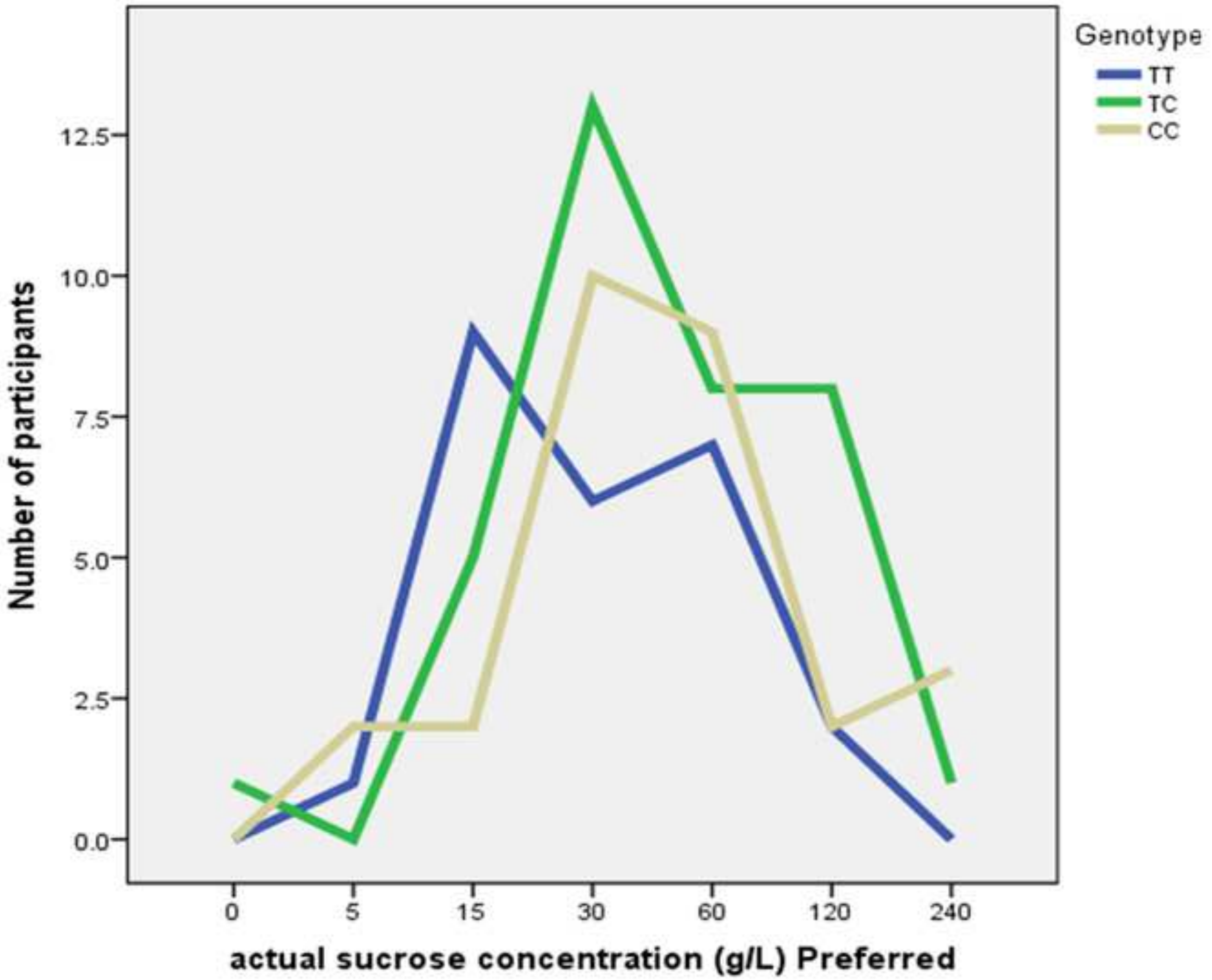

S1

Click here to access/download
Supporting Information
STROBE_checklist_cross-sectional.docx

S2

Click here to access/download
Supporting Information
data for analysis.xlsx
