# Supplementary material for: Gene variations and sweet taste sensitivity in Zambian adults with and without type 2 diabetes mellitus
Source: PLoS One. 2025 Jul 18;20(7):e0328172. doi: 10.1371/journal.pone.0328172 (PMC12273931; doi:10.1371/journal.pone.0328172)
Supplement: Attachment — Submitted filename: PONE-D-24-49147_R2 reviewer.pdf [file pone.0328172.s008.pdf]

To the Editors,

I hope this email finds you are well,

The manuscript has a minor revision. as some information in the genetic analysis part is confusing.

1- The paragraph: Collection of saliva samples and single nucleotide polymorphism (SNP) genotyping, please correct the dominant and recessive alleles for the TRPV1, as both the recessive allele and dominant allele are AC/CC, which is written as the following:

TRPV1; Transient Receptor Potential Cation Channel Subfamily V Member 1 gene, rs4790522; CC (wild type), AC, AA, AC/CC (recessive model), AC/CC (dominant model).

2- The reference 33, Pilic L, Mavrommatis Y. Genetic predisposition to salt-sensitive normotension 237 and its effects on salt taste perception and intake. Br J Nutr. 2018; 120:721–238 731. doi:10.1017/S0007114518002027 Is investigating the salty taste and the SNPs in the SLC4A5 (rs7571842, rs10177833), SCNN1B (rs239345), and TRPV1 (rs8065080) genes.

- The primers were not designed but used according to previous studies.
- Except the TRPV1 (rs8065080), it does cover the primers and the technique used for the study of TAS1R3 rs307355, and TRPV1 rs4790522

Thank you and best regards

Luma Al Obaidy

7-6-2025
