# Supplementary material for: Gene variations and sweet taste sensitivity in Zambian adults with and without type 2 diabetes mellitus
Source: PLoS One. 2025 Jul 18;20(7):e0328172. doi: 10.1371/journal.pone.0328172 (PMC12273931; doi:10.1371/journal.pone.0328172)
Supplement: Attachment — Submitted filename: Response to Reviewers.docx [file pone.0328172.s007.docx]

Please ensure that your manuscript meets PLOS ONE's style requirements, including those for file naming. The PLOS ONE style templates can be found at

<https://journals.plos.org/plosone/s/file?id=wjVg/PLOSOne_formatting_sample_main_body.pdf> and

[https://journals.plos.org/plosone/s/file?id=ba62/PLOSOne_formatting_sample_title_authors_affiliations.pdf](https://track.editorialmanager.com/CL0/https:%2F%2Fjournals.plos.org%2Fplosone%2Fs%2Ffile%3Fid=ba62%2FPLOSOne_formatting_sample_title_authors_affiliations.pdf/1/010f019384a3dd28-e9b45d46-c49f-47bc-b5fe-75cad20798ac-000000/4NQkxwwnyJZ_fF4NhtRcAmvE22lez4ObcBdWFtcdRE8=187)

**Response: Formatted as advised**

2. We note you have included a table to which you do not refer in the text of your manuscript. Please ensure that you refer to Table 2 in your text; if accepted, production will need this reference to link the reader to the Table.

**Response: The table has been cited**

3. Please include captions for your Supporting Information files at the end of your manuscript, and update any in-text citations to match accordingly. Please see our Supporting Information guidelines for more information: <http://journals.plos.org/plosone/s/supporting-information>.

**Response: Captions have been added for supporting information files**

**Comments to the Author**

1. Is the manuscript technically sound, and do the data support the conclusions?

The manuscript must describe a technically sound piece of scientific research with data that supports the conclusions. Experiments must have been conducted rigorously, with appropriate controls, replication, and sample sizes. The conclusions must be drawn appropriately based on the data presented.

Reviewer #1: Partly

Reviewer #2: Yes

**Response: we have made adjustments to ensure that the manuscript support the conclusions**

2. Has the statistical analysis been performed appropriately and rigorously?

Reviewer #1: Yes

Reviewer #2: Yes

**Response: Thank you**

3. Have the authors made all data underlying the findings in their manuscript fully available?

The [PLOS Data policy](http://track.editorialmanager.com/CL0/http:%2F%2Fwww.plosone.org%2Fstatic%2Fpolicies.action%23sharing/1/010f019384a3dd28-e9b45d46-c49f-47bc-b5fe-75cad20798ac-000000/-STxYFyVJMwbPCjgYdGOFmR0c8P_uj4g0oxL1BPyUi0=187) requires authors to make all data underlying the findings described in their manuscript fully available without restriction, with rare exception (please refer to the Data Availability Statement in the manuscript PDF file). The data should be provided as part of the manuscript or its supporting information, or deposited to a public repository. For example, in addition to summary statistics, the data points behind means, medians and variance measures should be available. If there are restrictions on publicly sharing data—e.g. participant privacy or use of data from a third party—those must be specified.

Reviewer #1: Yes

Reviewer #2: Yes

**Response: Thank you**

4. Is the manuscript presented in an intelligible fashion and written in standard English?

PLOS ONE does not copyedit accepted manuscripts, so the language in submitted articles must be clear, correct, and unambiguous. Any typographical or grammatical errors should be corrected at revision, so please note any specific errors here.

Reviewer #1: Yes

Reviewer #2: Yes

**Response: Thank you**

5. Review Comments to the Author

Reviewer #1: The manuscript has low quality. It did not address any specific gap they intended to fill. Furthermore, it does not add anything to the subject area compared with other published Material. Therefore, any comments regarding the methodology are equivocal. In addition, the discussion was inappropriate. This manuscript does not fulfill the standards established for the journal to be considered for publication.

**Response: We appreciate the reviewers comment. We have added more information to address the specific gap and have clearly written out the main goal of the study. Our study is unique in this population and we present novel results. One main reason for the apparent discrepancy with published material is because the black African population is genetically different so much that there are variations in SNPs. We think that our study will provide good baseline data for future studies that build on this field of study. Further, there are few studies which have investigated taste sensitivity and measured these genetic SNPs in African populations. We did a thorough search of literature as reported in the introduction and discussion to this effect and found no specific study.**

**We went ahead to add more SNPs to provide more relevance to the study. The limitation here is that the SNPs that may be associated with taste sensitivity in Caucasian populations where these studies are conducted may not necessarily be similar to our population. Measuring a wide ray of SNPs would be better in identifying SNPs common to African populations but the cost implications are prohibiting and beyond the scope of our study.**

Reviewer #2: The Manuscript sounds ok according to the number of participants. I think sample size is small. But its ok but you should add more case studies to prove the data and add more references and improve little bit your English.

**Response: We want to thank the reviewer for the suggestions. We have now added more single nucleotide polymorphisms to our data to enrich the manuscript and provided more clearer aims. We have also made editions to the grammar throughout to improve the manuscript. In addition, refer to our response to reviewer 1 for additional changes we have made to improve the manuscript**
