# Supplementary material for: Gene variations and sweet taste sensitivity in Zambian adults with and without type 2 diabetes mellitus
Source: PLoS One. 2025 Jul 18;20(7):e0328172. doi: 10.1371/journal.pone.0328172 (PMC12273931; doi:10.1371/journal.pone.0328172)
Supplement: Attachment — Submitted filename: Letter to Editor_Author Comments.docx [file pone.0328172.s009.docx]

Dear editor,

Thank you for the communication and consideration of our manuscript. We have spent time to make improvements aligned with both reviewers’ comments and have responded in a point-counter-point manner below. We hope that you find the revised manuscript appropriate for publication.

**Reviewer #3: The research question is interesting, and the methodology employed is generally appropriate. Additionally, the manuscript adheres to the general standards of scientific writing. However, there are some critical points that require attention to align with the publication standards of PLOS ONE.**

We thank you for seeing value in our manuscript and hope that the revisions made align with the publication standard of PLOS ONE.

**Limitations:
The authors mention many constraints, even thought you discussion of the limitations—such as potential biases, confounding variables, and the lack of significant results— enhance the transparency and rigor of the study.**

**Suggestions for Improvement:**

**Addressing Sample Size Limitations: If feasible, increasing the sample size or discussing why the current size is appropriate given the study design would improve the manuscript.**

Unfortunately, we are unable to increase our sample size, this is due to funding restraints. We hope that the publication of our manuscript will warrant further interest in researching our cohort and encourage future studies, including our own, to achieve a larger sample size. We have referenced our sample size limitations throughout the discussion from line.

**Supplementary Analysis: Including additional analyses (such as dominant or recessive model in statistical analysis, odds ratio) could help increase the robustness of the findings.**

We have now included additive, dominant and recessive models throughout the manuscript. We also have included a genotype grouping related to those carrying homozygous alternate alleles of the genes studied. We believe this suggested improvement has strengthened our manuscript so we would like to thank you for the consideration. In addition, we have assessed the sample size limitations we have when working with such a novel cohort, and as a result have decided to remove the TRPV1 rs8065080 SNP as the minor allele is not represented enough in our cohort. We believe this makes our reported findings stronger for the remaining two genotypes.

The manuscripts results and conclusion have been extensively revise upon your comment, so we hope that you find this version suitable for publication.

**Reviewer #4: Reviewer #1: Summary:**

**Mwakyoma et al. investigated the relationship between genetic variations in TRPV1 (rs4790522, rs8065080) and TAS1R3 (rs307355) genes and sweet taste sensitivity in Zambian adults with and without type 2 diabetes mellitus (T2DM). Using a cross-sectional design (89 participants: 47 T2DM, 42 non-di2DM), they found reduced sweet taste sensitivity in T2DM individuals but no association between the studied SNPs and taste thresholds. The study highlights the need for larger cohorts and emphasizes potential non-genetic factors influencing taste perception in T2DM.**

We thank you for seeing value in our manuscript and agree regarding the necessity to encourage further research in African cohorts.

**Overall Impression
The study addresses an under-researched topic in an African population, providing novel baseline data. However, limitations in sample size and methodology reduce the generalizability of conclusions. The relatively small sample size in this study constrained the depth of subsequent analyses. However, given the availability of large-scale GWAS datasets from diverse ethnic populations (e.g., European and East Asian cohorts), the authors could further strengthen the findings through two complementary approaches:

1.Implementing Mendelian randomization (MR) analyses to investigate potential causal relationships between sweet taste sensitivity and T2DM across distinct ethnic groups;

2.Conducting cross-ancestry comparative analyses to evaluate whether the lead SNP effects/alleles associated with these traits are conserved between African populations and other ethnicities. Such trans-ethnic validation would enhance the generalizability of the findings while addressing population-specific genetic architectures.**

We thank you for providing some methodological alternatives and agree that we are working with a small sample size. However, unfortunately we are unable to increase our sample size nor utilise other databases for this current study. This is due to funding restrains (including personnel). However, we have adapted our approach based on an alternative reviewer’s suggestions, by incorporating an additive, dominant and recessive structure to our results section. We have also excluded one TRPV1 SNP due to poor representation of the alternate genotype. Our work seeks to explore group differences and associations only at this point, with the hope that publication will allow exposure of this under-research cohort leading to future, larger studies.

The manuscripts results and conclusion have been extensively revised upon the comments from both reviewers, so we hope that you find this version suitable for publication.
