# Supplementary material for: Gene variations and sweet taste sensitivity in Zambian adults with and without type 2 diabetes mellitus
Source: PLoS One. 2025 Jul 18;20(7):e0328172. doi: 10.1371/journal.pone.0328172 (PMC12273931; doi:10.1371/journal.pone.0328172)
Supplement: Attachment — Submitted filename: Rebuttal Letter_Round 2_June 2025.docx [file pone.0328172.s010.docx]

Dear Editor,

Thank you again for the communication. We have acknowledged both reviewers’ comments and have responded in a point-counter-point manner below. We hope that you find the revised manuscript appropriate for publication.

**Reviewer #5:**

We thank you for your careful overview of our manuscript. We have considered all comments are responded below.

1. **Addressing Age Confounding (Most Critical):**
   a. **In the Results/Discussion: More explicitly discuss the potential impact of the age difference on the observed taste sensitivity differences. Can you quantify how much taste sensitivity typically changes with age based on existing literature to contextualize the findings?**
   b. **Statistical (if feasible): Even if not for primary analysis, an exploratory age-adjusted analysis (such as ANCOVA for taste threshold with group as factor and age as covariate or stratifying by age if numbers permit) could provide some insight. If not feasible, state clearly why and reiterate this as a major caveat when interpreting the taste sensitivity difference between groups.**
a. Thank you for your comments. We have extended the paragraph in the discussion (paragraph 2; line 355-358) directly related to this result. We hope this provides more clarity. We believe we previously acknowledged the difference in age as a limitation in the discussion paragraph 1 and 6. We have therefore left these sections unchanged.
b. ANCOVA or similar analysis with a covariate is not appropriate with the sample size of this study. We previously acknowledged this in the limitations section (line 397-398). Upon your comment, we have extended our original sentence to highlight this further (line 398-399.
2. **Combined Genotype Finding (PwT2DM Preference):**
   a. **Clearly state the number of individuals in each subgroup for this specific analysis (those with high/low preferred concentration carrying the combined risk/non-risk alleles).**
   b. **Emphasize its exploratory nature due to the small sample size and potential for it being a chance finding, especially in the context of multiple implicit comparisons. Label it clearly as hypothesis-generating.**
a. Thank you for this comment, we believe it increases the quality of our manuscript. We have provided sample size in text (line: 282-283) and in Figures 3 and 5. New figures have been resubmitted with the manuscript.
b. The section in the results section that discusses the combined genetic results (line 388-395) highlights the limitations you have discussed in this feedback. We have extended the section with phrases recommended, for further emphasis.
3. **Discussion of "No Genetic Link":
   Continue to emphasize that the "no genetic link" refers specifically to the two SNPs studied in this cohort and under this study's power limitations. It does not rule out the involvement of these genes through other variants, gene-gene/gene-environment interactions, or the involvement of other unstudied taste-related genes.**

After an in-depth discussion with the authors, we have concluded that line 85-102 and 376-395 emphasise than many studies have shown candidate and polygenic genetic associations with taste. Thus, we have not amended the introduction or discussion. However, we thank you for indicating this phrasing in the abstract and have updated the results and conclusion accordingly (line: 42-53).

4. **Clarity in Figures/Tables:
   Ensure all figures and tables clearly state the 'n' for each group/subgroup being compared, especially for genotype-based analyses. S1 Table 1 (combined genotypes for healthy) and the similar analysis for PwT2DM (Table 3 combined analysis leading to Fig 5) should clearly show participant numbers in each combined genotype category.**

Supplementary Table 1 already includes the sample size for genotype groupings, if we have misunderstood your comment please advise. As per feedback comment 2 we have updated Figure 3 & 5 accordingly. We have also updated Figures 2,3 and 6 accordingly.

5. **Future Directions (already good but could be slightly expanded):**
   a. **Reiterate the need for larger, ideally age-matched, cohort studies in diverse African populations.**
   b. **Suggest investigating a wider panel of taste-related genes or using hypothesis-free approaches (e.g., GWAS, if feasible in the future for such cohorts) to identify novel variants associated with taste perception and T2DM risk in African populations.**
   c. **The paradox of lower sensitivity but lower preference in PwT2DM is intriguing. Future studies could explore this with detailed dietary records, psychological assessments of food reward/craving, and awareness of dietary recommendations.**

Thank you for the kind words related to our final section. We have discussed you feedback thoroughly and amended the conclusion section, specifically related to point a and b. We hope that you consider this expansion ready for publication.

**Reviewer #6:**

We thank you for your careful overview of our manuscript. We have considered all comments are responded below.

**Please correct this information: 1- The paragraph: Collection of saliva samples and single nucleotide polymorphism (SNP) genotyping. Please correct the dominant and recessive alleles for the TRPV1, as both the recessive allele and dominant allele are AC/CC, which is written as the following: TRPV1; Transient Receptor Potential Cation Channel Subfamily V Member 1 gene, rs4790522; CC (wild type), AC, AA, AC/CC (recessive model), AC/CC (dominant model).**

Thank you for spotting this typo, it has been corrected (line 192).

**2- The reference 33: Pilic L, Mavrommatis Y. Genetic predisposition to salt-sensitive normotension 237 and its effects on salt taste perception and intake. Br J Nutr. 2018; 120:721–238 731. doi:10.1017/S0007114518002027, Is investigating the salty taste and the SNPs in the SLC4A5 (rs7571842, rs10177833), SCNN1B (rs239345) and TRPV1 (rs8065080) genes.**

- **The primers were not designed but used according to previous studies.**
- **Except the TRPV1 (rs8065080), it does cover the primers and the technique used for the study of TAS1R3 rs307355, and TRPV1 rs4790522**

Reference 33 is related to the genotyping methodology (extraction, quantification, genotyping), not the assay/primer design. We have revised the section titled “*Collection of saliva samples and single nucleotide polymorphism (SNP) genotyping”,* which we hope better portrays this (line 158-181).

Kind regards,

The authors
